# Return on Investment for Digital Behavioral Counseling in Patients With Prediabetes and Cardiovascular Disease

**DOI:** 10.5888/pcd13.150357

**Published:** 2016-01-28

**Authors:** Wenqing Su, Fang Chen, Timothy M. Dall, William Iacobucci, Leigh Perreault

**Affiliations:** Author Affiliations: Fang Chen, Timothy M. Dall, William Iacobucci, IHS Life Sciences, Washington, DC; Leigh Perreault, University of Colorado Anschutz Medical Campus, Aurora, Colorado.

## Abstract

**Introduction:**

We calculated the health and economic impacts of participation in a digital behavioral counseling service that is designed to promote a healthful diet and physical activity for cardiovascular disease prevention in adults with prediabetes and cardiovascular disease risk factors (Prevent, Omada Health, San Francisco, California). This program enhances the Centers for Disease Control and Prevention’s Diabetes Prevention Recognition Program. Participants completed a 16-week core program followed by an ongoing maintenance program.

**Methods:**

Analysis was conducted for 2 populations meeting criteria for lifestyle intervention: 1) prediabetes (n = 1,663), and 2) high cardiovascular disease risk (n = 2,152). The Markov-based model simulated clinical and economic outcomes related to obesity and diabetes annually over 10 years for the 2 defined populations. Comparisons were made between participants and propensity-matched controls from the community.

**Results:**

The return-on-investment break-even point was 3 years in both populations. Simulated return on investment for the population with prediabetes was $9 and $1,565 at years 3 and 5, respectively. Simulated return on investment for the population with cardiovascular disease risk was $96 and $1,512 at years 3 and 5, respectively. Results suggest that program participation reduces diabetes incidence by 30% to 33% and stroke by 11% to 16% over 5 years.

**Conclusion:**

Digital Behavioral Counseling provides significant health benefits to patients with prediabetes and cardiovascular disease and a positive return on investment.

## Introduction

One in 3 adults in the United States has prediabetes (fasting blood glucose, 100–125 mg/dL); 15% to 30% of those adults will develop type 2 diabetes mellitus within 5 years (1). Diabetes is associated with an additional $7,900 in average annual medical costs and with increased risk of cardiovascular disease (2).

The Diabetes Prevention Program (DPP) was a large clinical trial that showed that lifestyle intervention to improve diet and physical activity reduced type 2 diabetes mellitus onset by 58% over 3 years, with continued clinical benefits observed at 10-year and 15-year follow-up (3–5). In part because of the DPP results, the US Preventive Services Task Force (USPSTF) issued recommendations that overweight or obese adults with at least 1 other risk factor for cardiovascular disease receive behavioral counseling (6).

Given the success of the DPP, the Centers for Disease Control and Prevention (CDC) Diabetes Prevention Recognition Program standards were created to track and ensure the quality of lifestyle intervention programs aimed at diabetes risk reduction, including guidance for technology-based delivery of lifestyle interventions (7). Programs like the one we modeled here meet or exceed the Diabetes Prevention Recognition Program standards by using digital tools such as scales and pedometers connected to systems through the Internet, curricula that are accessible online and through mobile devices, and online support groups moderated by health coaches. The contribution of our study is the estimated return on investment for adults voluntarily participating in a digital behavioral counseling program, calculated by simulating clinical and economic outcomes for patients with prediabetes and additional cardiovascular disease risk factors.

## Methods

We used a previously published microsimulation model of the economic and clinical benefits of disease prevention (8–10) to calculate return on investment associated with participation in a digital behavioral counseling program (with 2 components, Prevent and Sustain, Omada Health, San Francisco, California). We applied 26-week weight loss results to simulate potential health and economic outcomes over the subsequent 10 years on 2 populations defined by prediabetes status and presence of other cardiovascular disease risk factors.

Published results from a pilot study of Prevent analyzed 187 participants who started the program and met eligibility criteria, including a previous diagnosis of prediabetes (11). Average weight of participants declined 5.0% at 16 weeks and 4.8% at 12 months, which was accompanied by an average 0.4% decrease in hemoglobin A1c (HbA1c). The 144 participants who continued into the Sustain phase experienced an average weight loss of 5.4% at week 16 and 5.2% at 12 months, as well as a 0.4% reduction in HbA1c after 12 months (11).

To calculate return on investment, the average program cost for an active participant was estimated at $1,300 over 3 years, including an up-front cost of $400 for weight loss courses and $900 for the Sustain program over the following 3 years ($400 at the end of year 1, $300 at the end of year 2, and $200 at the end of year 3).

### Model population

The study sample consisted of 2,371 people enrolled in Prevent between 2012 and 2014 and in the program for at least 16 weeks, older than 18 years, and with baseline body mass index (BMI) of 24 kg/m^2^ or higher. We calculated the difference of each participant’s beginning body weight and last available weight recorded between week 17 and 26.

Prevent collects BMI and demographic data on participants but does not collect additional clinical and health risk information required by the computer model to simulate disease onset and medical expenditures or to determine whether participants have prediabetes or cardiovascular disease risk factors. Required information for modeling includes HbA1c, systolic blood pressure (SBP) and diastolic blood pressure (DBP), total cholesterol and high-density lipoprotein cholesterol (HDL-C), current smoking status, and presence of chronic medical conditions (eg, treated or untreated hypertension, diabetes, cardiovascular disease, and history of myocardial infarction or stroke). To extrapolate these missing variables, we performed a 1:1 propensity score match (on age, sex, race/ethnicity, and starting BMI) with the 2011–2012 National Health and Nutrition Examination Survey (NHANES) population.

Diabetes Prevention Recognition Program enrollment criteria include adults with a BMI of 24 kg/m^2^ or higher and at least 50% of program participants have diagnosed prediabetes or history of diagnosed gestational diabetes (7). USPSTF recommends behavioral counseling for overweight or obese adults with 1 or more additional risk factors for cardiovascular disease (6). On the basis of propensity matching with NHANES, the demographics and BMI of Prevent participants suggested that if a random sample of overweight and obese adults participated, then approximately 53% would have prediabetes and 77% would meet the USPSTF criteria of having at least 1 risk factor for cardiovascular disease.

Our study focused on the populations with prediabetes and populations at risk for cardiovascular disease (because those populations are recommended to receive behavioral counseling), although we also analyzed the entire overweight and obese population for comparison. Correspondingly, we derived and analyzed 3 populations:

1. Population with prediabetes. Using propensity scoring, we matched 1,663 Prevent participants to a person in NHANES with prediabetes (HbA1c 5.7%–6.4%) and the same demographics and BMI.
2. Cardiovascular disease risk population. We matched 2,152 Prevent participants to adults in NHANES with the same demographics and BMI and who had at least 1 cardiovascular disease risk factor.
3. Overweight and obese population. We matched all 2,371 Prevent participants to an NHANES person with the same demographics and BMI.

Prevent participants not matched through propensity scoring were predominantly young women with high BMI for whom there were few similar adults in NHANES.

The starting (time 0) characteristics of these analytic cohorts are summarized in Table 1. These participants constitute the intent-to-treat cohorts. We also analyzed starters (participants completing ≥4 lessons) and completers (completing ≥9 lessons) according to CDC guidelines (7).

**Table 1:** Characteristics of Population With Prediabetes and Population at Risk for Cardiovascular Disease at Baseline, Prevent Digital Behavioral Counseling Program, 2012–2014^a^

Patient Characteristic	Population With Prediabetes			US Preventive Services Task Force Population		
	Total (N = 1,663)	Starters^b^ (N = 1,462)	Completers^c^ (N = 1,232)	Total (N = 2,152)	Starters^b^ (N = 1,892)	Completers^c^ (N = 1,588)
Age, mean (SE), y	49.8 (0.30)	50.6 (0.31)	51.7 (0.33)	49.0 (0.27)	50.9 (0.27)	50.9 (0.29)
BMI, mean (SE), kg/m^2^	33.9 (0.15)	33.9 (0.16)	33.7 (0.17)	34.4 (0.14)	34.0 (0.14)	34.0 (0.16)
SBP, mean (SE), mm Hg	126.8 (0.43)	126.7 (0.46)	126.5 (0.50)	126.3 (0.41)	126.4 (0.41)	126.4 (0.45)
HDL-C, mean (SE), mg/dL	49.8 (0.34)	49.8 (0.36)	49.6 (0.39)	50.3 (0.34)	50.5 (0.34)	50.5 (0.38)
T-C, mean (SE), mg/dL	201.3 (0.97)	200.9 (1.02)	200.3 (1.12)	203.0 (1.00)	203.0 (1.00)	203.0 (1.09)
Hemoglobin A1c, mean (SE), %	5.9 (0.01)	5.9 (0.01)	5.9 (0.01)	6.0 (0.02)	6.0 (0.02)	6.0 (0.03)
Male, %	30.8	30.1	31.7	24.1	24.8	24.8
Prediabetes, %	100	100	100	62.6	62.8	62.8
Diabetes, %	0	0	0	17.1	16.8	16.8

Abbreviations: BMI, body mass index; HDL-C, high-density lipoprotein cholesterol; SBP, systolic blood pressure; SE, standard error; T-C, total cholesterol.

a All values estimated from a population with individuals matched from the National Health and Nutrition Examination Survey.

b Participants completing ≥4 lessons.

c Participants completing ≥9 lessons.

### Simulation modeling

We used a Markov-based microsimulation model in which a person’s characteristics are used to predict health outcomes in the upcoming year. Detailed documentation of the model prediction equations for disease onset and mortality, data and assumptions that underlie the model, calculations for quality-adjusted life years (QALYs), and validation results are published elsewhere (8,9).

The simulation model uses an annual cycle, with weight loss occurring at time 0, after which body weight follows a natural history of weight change (increasing or decreasing) as a person ages (8,9). Change in body weight affects HbA1c, blood pressure, and cholesterol levels, and these clinical outcomes combined with demographics, smoking status, and presence of chronic conditions in turn are used in prediction equations for disease onset, severity, and mortality. Equations predicting annual disease states are based on published clinical and observational studies (8,9). Simulated annual medical expenditures, estimated with the 2008–2012 Medical Expenditure Panel Survey by using a generalized linear model with gamma distribution and log link, reflect patient demographics; presence of diabetes, hypertension, congestive heart failure, ischemic heart disease, retinopathy, and end-stage renal disease; history of myocardial infarction, stroke, and various cancers; smoking status; and body weight. Regressions results are published elsewhere (9).

To fully account for real-life uncertainty in the effectiveness of weight loss programs, each person’s actual 26-week weight loss was used in the simulation. All medical costs were converted to 2014 dollars by using the medical component of the consumer price index (12). Dollar estimates are in 2014 US dollars (using 3% discount rate) unless otherwise indicated.

Construction and validation of the model followed recommendations from the International Society for Pharmacoeconomics and Outcomes Research for best practices and transparency (13,14). Validation activities included review by experts in obesity, endocrinology, modeling, and health economics; internal and external quantitative validation (8,9); and sensitivity analysis to test the robustness of model conclusions under additional uncertainties. As part of the sensitivity analysis, we simulated outcomes if each person’s weight loss varied by ±1 percentage point (absolute change) relative to actual observed weight loss. For example, a person who experienced 4.5% weight loss was also modeled with 3.5% and 5.5% weight loss. Because the average weight loss of Prevent participants was about 5%, a ±1 percentage-point sensitivity analysis is equivalent to ±20% (relative change) around baseline weight loss and simulates the implications if average weight loss were approximately 4% to 6% (a range observed for other lifestyle intervention programs).

## Results

### Population with prediabetes

The constructed population with prediabetes was on average aged 49.8 years (standard error [SE], 0.30 y), and 30.8% were men (Table 1). The average starting BMI was 33.9 kg/m^2^ (SE, 0.15 kg/m^2^), and HbA1c was 5.9% (SE, 0.01%). Starter and completer subgroups were similar to the overall intent-to-treat cohort.

On average, participants with prediabetes lost 5.1% of body weight. The simulated reduction in diabetes onset was 28%, 30%, and 26% over 3, 5, and 10 years, respectively (Table 2). Participants with a history of cardiovascular disease–related events showed a modest 9% to 17% decrease for additional cardiovascular disease events, and simulated incidence of stroke declined 16% over 5 years. Simulation results suggest reduced cumulative medical expenditures over 3, 5, and 10 years by, $1,310, $2,865, and $9,217, respectively.

**Table 2:** Simulated Outcomes for Population With Prediabetes and Subgroups, Prevent Digital Behavioral Counseling Program, 2012–2014

Outcome	Intent-to-Treat			Starters^a^			Completers^b^		
	3-year	5-year	10-year	3-year	5-year	10-year	3-year	5-year	10-year
**Disease onset relative reduction, %**									
Diabetes	27.9	29.5	26.0	31.4	32.4	28.0	33.3	36.1	31.2
History of ischemic heart disease	9.1	12.7	14.2	9.9	11.9	12.3	16.6	15.1	14.5
History of myocardial infarction	11.9	10.9	16.3	5.9	10.5	15.9	16.2	17.2	20.1
History of congestive heart failure	14.6	15.0	15.4	13.1	14.9	14.7	15.3	16.6	17.6
History of stroke	15.2	16.3	15.7	15.1	16.1	15.5	21.3	21.8	18.7
Obstructive sleep apnea	9.6	10.0	9.8	11.2	10.7	10.3	11.7	12.1	11.4
Major depression episodes	9.0	10.0	10.9	11.0	11.5	12.4	12.0	13.6	14.4
**Medical expenditures saving, $ per capita^c^**	1,310	2,865	9,217	1,533	3,317	10,043	1,790	3,893	12,026
**QALYs increase per person**	0.05	0.10	0.23	0.06	0.11	0.26	0.06	0.12	0.28
**Sick days reduction per person**	0.4	0.7	1.6	0.4	0.8	2.0	0.5	0.8	1.8
**Average 3-year program costs, $^c^**	1,300								
**Average weight loss, %**	5.1			5.3			6.0		
**Sample size, n**	1,663			1,462			1,232		

Abbreviation: QALYs, quality-adjusted life years.

a Participants completing ≥4 lessons.

b Participants completing ≥9 lessons.

c Dollar estimates are 2014 US dollars value using a 3% discount rate.

Average weight loss (recorded on or before week 26) among program completers (6.0%) exceeded average weight loss among starters (5.3%) and the intent-to-treat cohort (5.1%). Both groups had simulated positive return on investment within 3 years. Simulated cumulative medical savings over 3, 5, and 10 years averaged $1,533, $3,317, and $10,043 for program starters and $1,730, $3,893, and $12,026 for program completers.

### Population at risk for cardiovascular disease

The population meeting USPSTF screening criteria was on average aged 49.0 years (SE, 0.27 y), 24.1% were male, and average starting BMI was 34.4 kg/m^2^ (SE, 0.14 kg/m^2^) (Table 1). An estimated 62.6% had prediabetes, 17.1% had diabetes, and 20.3% had normal blood glucose levels.

Weight loss among this analytic cohort averaged 5.0%. Simulated average reductions in cumulative medical expenditures were $1,396, $2,812, and $7,951 over 3, 5, and 10 years, respectively (Table 3). The average weight loss among starters and completers was 5.3% and 6.0%, respectively. Simulated medical savings over 3, 5, and 10 years were higher for program completers versus program starters.

**Table 3:** Simulated Outcomes for US Preventive Services Task Force Population and Subgroups, Prevent Digital Behavioral Counseling Program, 2012–2014

Outcome	Intent-to-Treat			Starters^a^			Completers^b^		
	3-year	5-year	10-year	3-year	5-year	10-year	3-year	5-year	10-year
**Disease onset relative reduction, %**									
Diabetes	30.1	32.8	30.4	32.8	35.1	32.3	35.0	38.0	35.9
History of ischemic heart disease	10.6	9.3	9.2	10.4	11.1	13.0	11.9	13.1	13.1
History of myocardial infarction	11.8	9.0	14.0	8.2	10.6	13.7	11.3	12.2	16.7
History of congestive heart failure	8.4	9.4	10.5	6.9	10.4	12.5	12.0	10.9	12.3
History of stroke	11.2	11.1	13.1	11.1	12.5	12.4	17.7	15.1	15.1
Obstructive sleep apnea	11.9	11.6	10.9	11.3	11.0	10.9	10.9	10.2	10.3
Major depression episodes	9.7	9.2	10.5	10.5	10.8	11.9	12.4	11.7	13.2
**Medical expenditures saving, $ per capita^c^**	1,396	2,812	7,951	1,569	3,047	8,610	1,765	3,436	9,966
**QALYs increase per person**	0.05	0.10	0.21	0.06	0.11	0.23	0.07	0.12	0.26
**Sick days reduction per person**	0.5	0.7	1.8	0.5	0.8	1.7	0.5	0.9	2.1
**Average 3-year program costs, $^c^**	1,300								
**Average weight loss, %**	5.0			5.3			6.0		
**Sample size, n**	2,152			1,892			1,588		

Abbreviation: QALYs, quality-adjusted life years.

a Participants completing ≥4 lessons.

b Participants completing ≥9 lessons.

c Dollar estimates are 2014 US dollars value using a 3% discount rate.

### Return-on-investment analysis

**Figure 1:**
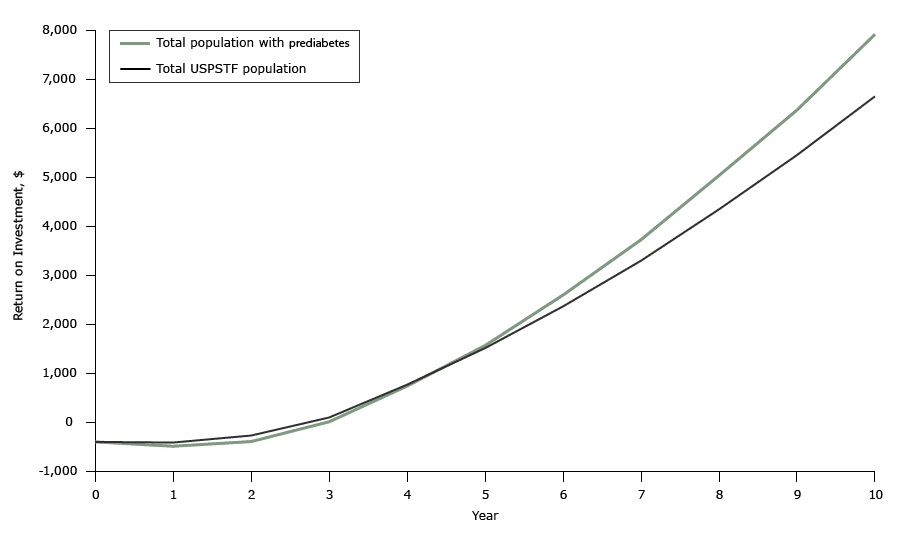
Projected average return on investment on weight loss program participation, Prevent digital behavioral counseling program, 2012–2014. Return on investment was calculated as the difference between the medical cost savings due to improvements in health and the cost of participating in the program (Return on investment = direct medical saving – Prevent initial program cost at year 0 – Prevent maintenance costs). Abbreviation: USPSTF, US Preventive Services Task Force. **Table:** 

In the intent-to-treat cohort, the projected break-even point was 3 years for both the population with prediabetes and the population with cardiovascular disease risk factors (Figure 1). At 3, 5, and 10 years, estimated return on investment averaged $9, $1,565, and $7,918 for the population with prediabetes and $96, $1,512, and $6,651 for the population at risk for cardiovascular disease. The percentage of return on investment in each year can be calculated by dividing “total return at or before current year” by “total investment at or before current year.” According to this algorithm, 39%, 64%, and 101% of program investment on the population with prediabetes was recouped at year 1, 2, and 3, respectively. For the population with risk factors for cardiovascular disease, 48%, 75%, and 107% of the investment was recouped at year 1, 2, and 3, respectively.

Year	Total Population With Prediabetes	Total USPSTF Population
0	-$400	-$400
1	-$490	-$415
2	-$396	-$273
3	$9	$96
4	$737	$766
5	$1,565	$1,512
6	$2,595	$2,364
7	$3,726	$3,297
8	$5,037	$4,344
9	$6,374	$5,455
10	$7,918	$6,651

## Discussion

Previous work found that Prevent is effective in reducing body weight and improving HbA1c levels among a population with prediabetes (11,15). Using microsimulation, we modeled the clinical and economic implications one would expect with this level of weight loss given the characteristics of program participants and whether participants had prediabetes or were at risk for developing cardiovascular disease.

**Figure 2:**
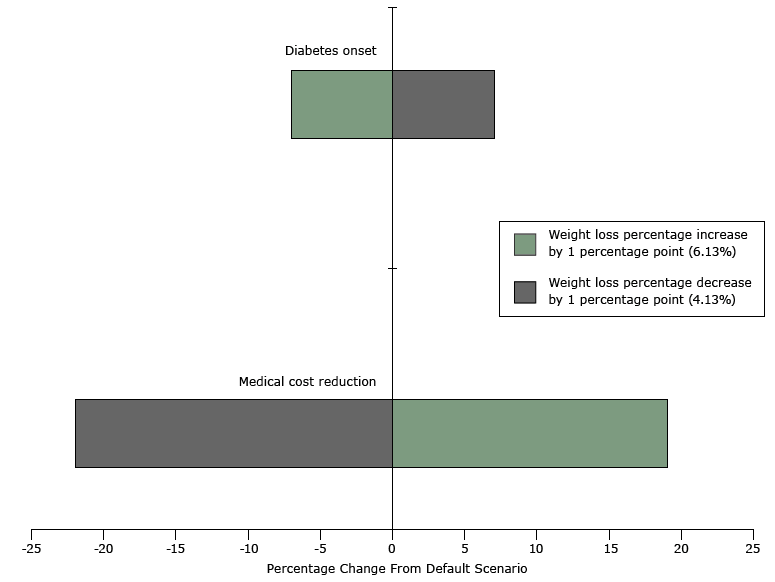
Tornado diagram for the sensitivity analysis on weight loss percentage over 10 years in a population with prediabetes, Prevent digital behavioral counseling program, 2012–2014. Default weight loss for the population with prediabetes is 5.13%; diabetes onset is based on the absolute number of new diabetes cases in the time period. **Table:** 

Given the central role of weight loss in the model, we conducted sensitivity analysis around weight loss to test its influence on program benefits. Findings were similar for the populations at risk for prediabetes and cardiovascular disease (Figure 2). In the population with prediabetes, simulated medical saving varied from −22% to 19%, and diabetes onset varied from −7% to 7%. Sensitivity analysis results on the population at risk for cardiovascular disease are in the Appendix.

Category	Weight loss percentage increase by 1 percentage point (6.13%)	Weight loss percentage decrease by 1 percentage point (4.13%)
Medical cost reduction	19%	−22%
Diabetes onset	−7%	7%

Study findings suggest that the 2014 US dollars value of reduced medical expenditures from achieving the average cumulative results of Prevent over 3, 5, and 10 years is, respectively, $1,310, $2,865, and $9,217. Among a broader population meeting USPSTF criteria, the savings are $1,396, $2,812, and $7,951 over 3, 5, and 10 years, respectively. Our findings agree with a CDC-initiated study, which concluded that implementing the USPSTF behavioral counseling recommendations can be cost effective (16). Certain subgroups in the general obese populations, such as the middle-aged population with class III obesity (BMI ≥40 kg/m^2^) and hypertension, could enjoy even higher savings in direct medical expenditures. Subgroup analysis in obese populations is a topic for future research.

Our findings are similar to previously published cost and disease estimates associated with implementing a lifestyle intervention in people with prediabetes — that achieving the average weight loss and HbA1c outcomes of the DPP trial could reduce cumulative medical expenditures by $10,600 over 10 years (2014 dollars, using a 3% discount rate), on average (8). A comparison of the outcomes from our microsimulation and those from the DPP and DPP Outcomes Study (DPPOS) has been detailed (8,9). One challenge of this comparison was that DPPOS was based on an obese population with many risk factors and a very high risk of developing type 2 diabetes. A second challenge was that after completion of the DPP study (at 3 years) all participants were offered the lifestyle intervention, thus diluting the potential long-term benefits in the DPPOS. However, our simulation results align with those of the study in the following ways. In the first 3 years, the benefits of lifestyle intervention were smaller in our simulation, which can be explained by a lower-risk population. Over 10 years, the benefits became larger — consistent with no cross-contamination of the intervention and control groups as occurred when the DPP control group later began the lifestyle intervention. One exception is reduction in diabetes onset after lifestyle intervention, which is lower in the simulation than in the DPP or DPPOS at both year 3 (28% vs 58%) and year 10 (26% vs 34%) (3,4).

We collected weight loss data for 26 weeks, and the return-on-investment analysis assumes that participants retain this weight loss with natural weight changes one would expect associated with aging (with average annual weight gain through approximately age 60). Findings from early Prevent participants found that after 2 years, a large proportion of the population has maintained their weight loss (15). Specifically, program completers lost an average 4.9% after 1 year and 4.3% after 2 years. The DPPOS found that in the 5 years following DPP lifestyle intervention there was gradual weight gain, with participants sustaining approximately one-third of their original weight loss between years 5 and 10 (4).

Prevent has an integrated 3-year Sustain component aiming at maintaining initial weight loss for an extended period of time. Nevertheless, we tested a scenario in which participants regain weight after year 1 at the same speed observed in DPPOS. Under this scenario, we found the return-on-investment break-even timeline increased by about 4 months and 10-year total savings dropped from $7,918 to $5,591 for the population with prediabetes.

Clinical trials and community-based programs have shown that lifestyle intervention can be effective in reducing body weight and improving health outcomes. An estimated 86 million adults have prediabetes, and many of these adults are candidates for lifestyle intervention (1). The population covered by the USPSTF recommendations for preventing cardiovascular disease is likely even larger. Treating a population this size requires alternative strategies to those tested in the in-person DPP. This study suggests that online programs may offer a scalable, cost-effective solution. Using Internet-based technologies can both help overcome geographic and scheduling barriers and allow participants to review material at their own pace.

Using a previously published and validated microsimulation model, we simulated how the clinical outcomes achieved by Prevent participants translate into reduced future prevalence of disease and reduced medical expenditures. Model strengths and limitations are discussed elsewhere in detail (8,9). Strengths include the ability to simulate outcomes over an extended period, using disease prediction equations based on published epidemiologic studies and accounting for the characteristics and outcomes of program participants. Limitations include the use of data from multiple sources (both US and non-US), older data such as those from the Framingham study when newer data were unavailable, and some disease onset predication equations based on a general population rather than a population with prediabetes or risk factors for cardiovascular disease. Additional limitations specific to this study include

1. Prevent participants chose to participate in the intervention. This means that results can be generalized to other populations of voluntary participants but not necessarily to the general population.
2. Weight loss data were the only clinical data collected on Prevent participants, so we used propensity matching (using age, sex, race/ethnicity, and BMI) with NHANES data to create the analytic files that contained all the starting values for clinical inputs needed to run the simulation. We could not directly observe if Prevent participants had prediabetes or risk factors for cardiovascular disease.
3. We were unable to find a match for 708 Prevent participants in the NHANES data. Unmatched people were overwhelmingly young women with much higher BMI than observed in NHANES participants. Unreported findings suggest that extremely obese people have higher return on investment from weight loss relative to less obese people, but younger people have lower short-term return on investment relative to older people.

DPP-based programs offered online can increase access to a cost-effective lifestyle intervention to millions of adults with prediabetes or who are at high risk for cardiovascular disease. In addition to improving health outcomes, such an intervention can provide a positive return on investment for payers.

## Appendix

This file is available for download as a Microsoft Word document.
