# Supplementary material for: Return on Investment for Digital Behavioral Counseling in Patients With Prediabetes and Cardiovascular Disease
Source: Prev Chronic Dis. 2016 Jan 28;13:E13. doi: 10.5888/pcd13.150357 (PMC4747438; doi:10.5888/pcd13.150357)
Supplement: Supplementary file 1 [file 15_0357_Appendix.docx]

# Appendix 1. Modeling Approach

## Overview

The model used to simulate the health and economic impact of Prevent is a Markov-based microsimulation model where a person’s current characteristics are used to predict health outcomes in the next year, with the process repeated annually for 10 years. In this section we provide an overview of the model; detailed technical documentation of the model data, methods, assumptions, and validation activities are published elsewhere (1). Construction and validation of the model followed recommendations from the International Society for Pharmacoeconomics and Outcomes Research for best practices and transparency (2,3). Validation activities include review by experts in obesity, endocrinology, modeling, and health economics; internal and external quantitative validation; and extensive sensitivity analysis. A schematic of the model is shown in Appendix Figure 1.

For each person in the sample we created a health risk profile that consisted of demographics (age, sex, race), the biometrics listed previously (SBP, DBP, A1c, HDL cholesterol, total cholesterol), current smoking status, and the presence of recognized obesity and diabetes-related comorbidities — hypertension, ischemic heart disease, congestive heart failure, renal failure, diabetic retinopathy, diabetic amputation, history of stroke, history of heart attack, history of 16 types of obesity-related cancers, major depression episodes, obstructive sleep apnea, and osteoarthritis (4,5).

## Clinical outcomes

Prediction equations that relate a person’s health risk factors to annual change in disease states came from published clinical and observational studies and are described in detail elsewhere (1). Annual changes in biometrics (SBP, DBP, total cholesterol and HDL cholesterol) were linked to age, sex, and BMI change using published parameters (1,6–8). For patients where diabetes onset was simulated, then prediction equations for change in biometrics and diabetes sequelae came from the United Kingdom Prospective Diabetes Study (UKPDS) Outcomes Model (9). Change in A1c levels was linked to age, change in BMI, and total cholesterol (1). Prediction equations for atrial fibrillation, left ventricular hypertrophy, ischemic heart disease, myocardial infarction, congestive heart failure, stroke, chronic kidney disease, renal failure, peripheral vascular disease, and diabetic retinopathy came from published sources (9–11). The primary data source for equations modeling incidence of cardiovascular disease and adverse events for people without diabetes was the Framingham Heart Study (8,12–14).

Although mortality probability was low among the population analyzed and there were few simulated deaths over 10 years among the sample analyzed, the simulation modeled all-cause and disease-specific annual mortality probability taking into account participant demographics, smoking status, and disease presence (9,15,16). The impact of disease and aging on quality of life reduction reflects published findings for a nationally representative sample of US adults (17,18).

We used each person’s health profile at intervention start (time 0) to simulate disease onset and change in biometrics over the first year (year 1). The simulated health risk profile for year 1 was used to simulate health outcomes for year 2, with the process repeated through year 10. In the absence of Prevent, this scenario represents the natural history of disease. Then, we modified each patient’s risk factors at time 0 to reflect his or her change in body weight and change in A1c (from the Pilot study) (19) achieved through Prevent and re-simulated outcomes reflecting the participants’ new health profile. Differences in simulated population health outcomes between the two scenarios (with and without Prevent) reflect the estimated health impact of weight loss and improved A1c associated with Prevent participation.

For the nonintervention scenario and for the intervention scenario for years 2 through 10, we modeled annual change in body weight associated with aging. The annual change in body weight associated with aging reflects average differences in BMI between subsequent ages using cross-sectional data with adults in the 2007–2008, 2009–2010, and 2011–2012 NHANES files. We estimated the relationship between age and BMI separately for men and women by BMI category (<25.0, 25.0–29.9, or ≥30.0). Validation activities found that this approach produced weight change patterns similar to published findings using longitudinal data (20).

## Economic outcomes

The model simulates annual medical expenditures based on demographics (age group, sex, race), the disease states modeled, smoking status, body weight status (normal weight, overweight, obese, and continuous BMI above 30). The prediction equation for medical expenditures came from generalized linear model with gamma distribution and log link, analyzing data from the 2006–2010 files (n = 165,913) of the Medical Expenditure Panel Survey (MEPS). Regression results are published elsewhere (1). Costs associated with cancer (onset year and subsequent year) came from published sources (21,22), as did costs for chronic kidney disease (23), gallstone disease (24), pneumonia (25), pulmonary embolism (26), osteoarthritis (27), gastroesophageal reflux disease (28), chronic back pain (29), and non-alcoholic fatty liver disease (30). These costs reflect annual costs for people living; the higher medical costs associated with end of life are based on published estimates (31).

Prediction equations for other economic outcomes (employment probability, earnings, missed work days, and receipt of Supplemental Security Income for disability) are based on regression analysis of 2008–2010 MEPS data linked with the National Health Interview Survey. Regression specifications and results are published elsewhere (1). The same explanatory variables described above were used, with employed status (n = 25,296) and receipt of Supplemental Security Income for disability (n = 26,080) both estimated by logistic regression and annual missed work days (n = 18,699) for employed adults analyzed using negative binomial regression. Ordinary least squares regression with MEPS data (n = 165,913) modeled household income. Probability of employment and household income was simulated through age 70.

## Appendix Figure 1. Model Overview


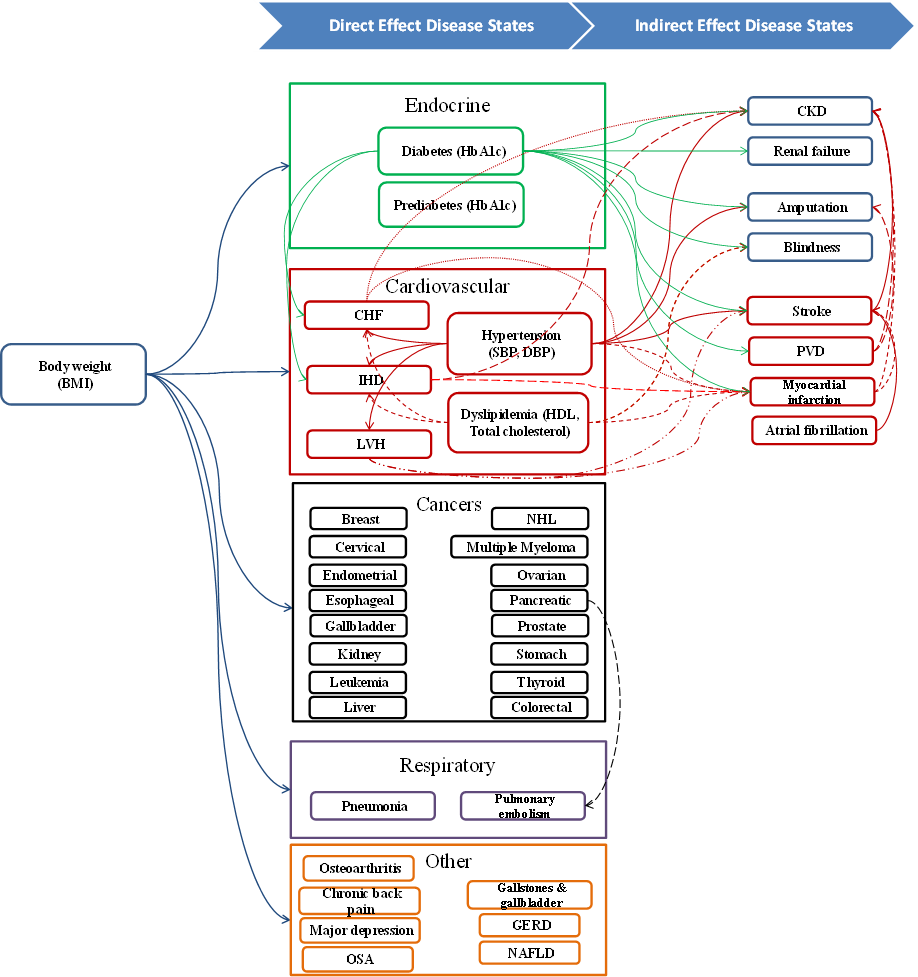


Note: Connecting lines show the items in the model that are linked. Abbreviations: BMI, body mass index; CHF, congestive heart failure; CKD, chronic kidney disease; DBP, diastolic blood pressure; GERD, gastroesophageal reflux disease; HbA1c, hemoglobin A1c; HDL, high-density lipoprotein; IHD, ischemic heart disease; LVH, left ventricular hypertrophy; NAFLD, nonalcoholic fatty liver disease; OSA, obstructive sleep apnea; PVD, peripheral vascular disease; SBP, systolic blood pressure.

## References

1. Dall TM, Storm MV, Semilla AP, Wintfeld N, O’Grady M, Narayan KMV. Value of lifestyle intervention to prevent diabetes and sequelae. Am J Prev Med 2015;48(3):271–80. <http://dx.doi.org/10.1016/j.amepre.2014.10.003> [PubMed](http://www.ncbi.nlm.nih.gov/entrez/query.fcgi?cmd=Retrieve&db=PubMed&list_uids=25498548&dopt=Abstract)

2. Eddy DM, Hollingworth W, Caro JJ, Tsevat J, McDonald KM, Wong JB; ISPOR−SMDM Modeling Good Research Practices Task Force. Model transparency and validation: a report of the ISPOR-SMDM Modeling Good Research Practices Task Force—7. Value Health 2012;15(6):843–50. <http://dx.doi.org/10.1016/j.jval.2012.04.012> [PubMed](http://www.ncbi.nlm.nih.gov/entrez/query.fcgi?cmd=Retrieve&db=PubMed&list_uids=22999134&dopt=Abstract)

3. Law AM. How to build valid and credible simulation models. In: Mason S, Hill R, Mönch L, Rose O, editors. Proceedings of the 40th Conference on Winter Simulation (WSC ’08); 2008 December 7–10; Miami, Florida.

4. American College of Cardiology/American Heart Association Task Force on Practice Guidelines, Obesity Expert Panel, 2013. Expert Panel Report: guidelines (2013) for the management of overweight and obesity in adults. Obesity (Silver Spring) 2014;22(S2, Suppl 2):S41–410. <http://dx.doi.org/10.1002/oby.20660> [PubMed](http://www.ncbi.nlm.nih.gov/entrez/query.fcgi?cmd=Retrieve&db=PubMed&list_uids=24227637&dopt=Abstract)

5. American Diabetes Association. Standards of medical care in diabetes — 2015. Diabetes Care 2015;38:S1–93. <http://care.diabetesjournals.org/content/suppl/2014/12/23/38.Supplement_1.DC1/January_Supplement_Combined_Final.6-99.pdf.%20> Accessed December 29, 2015.

6. Neter JE, Stam BE, Kok FJ, Grobbee DE, Geleijnse JM. Influence of weight reduction on blood pressure: a meta-analysis of randomized controlled trials. Hypertension 2003;42(5):878–84. <http://dx.doi.org/10.1161/01.HYP.0000094221.86888.AE> [PubMed](http://www.ncbi.nlm.nih.gov/entrez/query.fcgi?cmd=Retrieve&db=PubMed&list_uids=12975389&dopt=Abstract)

7. Heianza Y, Arase Y, Fujihara K, Hsieh SD, Saito K, Tsuji H, et al. Longitudinal trajectories of HbA1c and fasting plasma glucose levels during the development of type 2 diabetes: the Toranomon Hospital Health Management Center Study 7 (TOPICS 7). Diabetes Care 2012;35(5):1050–2. <http://dx.doi.org/10.2337/dc11-1793> [PubMed](http://www.ncbi.nlm.nih.gov/entrez/query.fcgi?cmd=Retrieve&db=PubMed&list_uids=22456865&dopt=Abstract)

8. Wilson PW, Anderson KM, Harris T, Kannel WB, Castelli WP. Determinants of change in total cholesterol and HDL-C with age: the Framingham Study. J Gerontol 1994;49(6):M252–7. <http://dx.doi.org/10.1093/geronj/49.6.M252> [PubMed](http://www.ncbi.nlm.nih.gov/entrez/query.fcgi?cmd=Retrieve&db=PubMed&list_uids=7963277&dopt=Abstract)

9. Clarke PM, Gray AM, Briggs A, Farmer AJ, Fenn P, Stevens RJ, et al.; UK Prospective Diabetes Study (UKDPS) Group. A model to estimate the lifetime health outcomes of patients with type 2 diabetes: the United Kingdom Prospective Diabetes Study (UKPDS) Outcomes Model (UKPDS no. 68). Diabetologia 2004;47(10):1747–59. <http://dx.doi.org/10.1007/s00125-004-1527-z> [PubMed](http://www.ncbi.nlm.nih.gov/entrez/query.fcgi?cmd=Retrieve&db=PubMed&list_uids=15517152&dopt=Abstract)

10. Nichols GA, Reinier K, Chugh SS. Independent contribution of diabetes to increased prevalence and incidence of atrial fibrillation. Diabetes Care 2009;32(10):1851–6. <http://dx.doi.org/10.2337/dc09-0939> [PubMed](http://www.ncbi.nlm.nih.gov/entrez/query.fcgi?cmd=Retrieve&db=PubMed&list_uids=19794003&dopt=Abstract)

11. Hippisley-Cox J, Coupland C. Predicting the risk of chronic kidney disease in men and women in England and Wales: prospective derivation and external validation of the QKidney Scores. BMC Fam Pract 2010;11(1):49. <http://dx.doi.org/10.1186/1471-2296-11-49> [PubMed](http://www.ncbi.nlm.nih.gov/entrez/query.fcgi?cmd=Retrieve&db=PubMed&list_uids=20565929&dopt=Abstract)

12. Wilson PW, Bozeman SR, Burton TM, Hoaglin DC, Ben-Joseph R, Pashos CL. Prediction of first events of coronary heart disease and stroke with consideration of adiposity. Circulation 2008;118(2):124–30. <http://dx.doi.org/10.1161/CIRCULATIONAHA.108.772962> [PubMed](http://www.ncbi.nlm.nih.gov/entrez/query.fcgi?cmd=Retrieve&db=PubMed&list_uids=18591432&dopt=Abstract)

13. Framingham Heart Study. Cardiovascular disease (10-year risk). https://www.framinghamheartstudy.org/risk-functions/cardiovascular-disease/10-year-risk.php. Accessed December 29, 2015.

14. US Department of Health and Human Services. The Framingham Study: an epidemiological intervention of cardiovascular diseases: section 34: some risk factors related to the annual incidence of cardiovascular disease and death using pooled repeated biennial measurements: Framingham Heart Study, 30 Year Followup. https://biolincc.nhlbi.nih.gov/static/studies/framcohort/30-Year_Followup_(Section_34).pdf. Accessed December 29, 2015.

15. Schaufelberger M, Swedberg K, Köster M, Rosén M, Rosengren A. Decreasing one-year mortality and hospitalization rates for heart failure in Sweden; data from the Swedish Hospital Discharge Registry 1988 to 2000. Eur Heart J 2004;25(4):300–7. <http://dx.doi.org/10.1016/j.ehj.2003.12.012> [PubMed](http://www.ncbi.nlm.nih.gov/entrez/query.fcgi?cmd=Retrieve&db=PubMed&list_uids=14984918&dopt=Abstract)

16. Centers for Disease Control and Prevention, NCHS. Underlying cause of death 1999–2010 on CDC WONDER online database, released 2012. Centers for Disease Control and Prevention, National Center for Health Statistics; 2013; Accessed July 1, 2013.

17. Zhang P, Brown MB, Bilik D, Ackermann RT, Li R, Herman WH. Health utility scores for people with type 2 diabetes in U.S. managed care health plans: results from Translating Research Into Action for Diabetes (TRIAD). Diabetes Care 2012;35(11):2250–6. <http://dx.doi.org/10.2337/dc11-2478> [PubMed](http://www.ncbi.nlm.nih.gov/entrez/query.fcgi?cmd=Retrieve&db=PubMed&list_uids=22837369&dopt=Abstract)

18. Sullivan PW, Lawrence WF, Ghushchyan V. A national catalog of preference-based scores for chronic conditions in the United States. Med Care 2005;43(7):736–49. <http://dx.doi.org/10.1097/01.mlr.0000172050.67085.4f> [PubMed](http://www.ncbi.nlm.nih.gov/entrez/query.fcgi?cmd=Retrieve&db=PubMed&list_uids=15970790&dopt=Abstract)

19. Sepah SC, Jiang L, Peters AL. Translating the Diabetes Prevention Program into an online social network: validation against CDC Standards. Diabetes Educ 2014;40(4):435–43. <http://dx.doi.org/10.1177/0145721714531339> [PubMed](http://www.ncbi.nlm.nih.gov/entrez/query.fcgi?cmd=Retrieve&db=PubMed&list_uids=24723130&dopt=Abstract)

20. Sheehan TJ, DuBrava S, DeChello LM, Fang Z. Rates of weight change for black and white Americans over a twenty year period. Int J Obes Relat Metab Disord 2003;27(4):498–504. <http://dx.doi.org/10.1038/sj.ijo.0802263> [PubMed](http://www.ncbi.nlm.nih.gov/entrez/query.fcgi?cmd=Retrieve&db=PubMed&list_uids=12664083&dopt=Abstract)

21. National Cancer Institute. Annualized mean net costs of care. 2011. http://costprojections.cancer.gov/annual.costs.html#f1. Accessed December 29, 2015.

22. Yabroff KR, Lamont EB, Mariotto A, Warren JL, Topor M, Meekins A, et al. Cost of care for elderly cancer patients in the United States. J Natl Cancer Inst 2008;100(9):630–41. <http://dx.doi.org/10.1093/jnci/djn103> [PubMed](http://www.ncbi.nlm.nih.gov/entrez/query.fcgi?cmd=Retrieve&db=PubMed&list_uids=18445825&dopt=Abstract)

23. United States Renal Data System. Chapter 7. Costs of chronic kidney disease. 2014. http://www.usrds.org/2012/view/v1_07.aspx. Accessed December 29, 2015.

24. Glasgow RE, Cho M, Hutter MM, Mulvihill SJ. The spectrum and cost of complicated gallstone disease in California. Arch Surg 2000;135(9):1021–5, discussion 1025–7. <http://dx.doi.org/10.1001/archsurg.135.9.1021> [PubMed](http://www.ncbi.nlm.nih.gov/entrez/query.fcgi?cmd=Retrieve&db=PubMed&list_uids=10982504&dopt=Abstract)

25. Colice GL, Morley MA, Asche C, Birnbaum HG. Treatment costs of community-acquired pneumonia in an employed population. Chest 2004;125(6):2140–5. <http://dx.doi.org/10.1378/chest.125.6.2140> [PubMed](http://www.ncbi.nlm.nih.gov/entrez/query.fcgi?cmd=Retrieve&db=PubMed&list_uids=15189934&dopt=Abstract)

26. Park B, Messina L, Dargon P, Huang W, Ciocca R, Anderson FA. Recent trends in clinical outcomes and resource utilization for pulmonary embolism in the United States: findings from the nationwide inpatient sample. Chest 2009;136(4):983–90. <http://dx.doi.org/10.1378/chest.08-2258> [PubMed](http://www.ncbi.nlm.nih.gov/entrez/query.fcgi?cmd=Retrieve&db=PubMed&list_uids=19525357&dopt=Abstract)

27. Kotlarz H, Gunnarsson CL, Fang H, Rizzo JA. Insurer and out-of-pocket costs of osteoarthritis in the US: evidence from national survey data. Arthritis Rheum 2009;60(12):3546–53. <http://dx.doi.org/10.1002/art.24984> [PubMed](http://www.ncbi.nlm.nih.gov/entrez/query.fcgi?cmd=Retrieve&db=PubMed&list_uids=19950287&dopt=Abstract)

28. Bloom BS, Jayadevappa R, Wahl P, Cacciamanni J. Time trends in cost of caring for people with gastroesophageal reflux disease. Am J Gastroenterol 2001;96(8, Suppl):S64–9. <http://dx.doi.org/10.1016/S0002-9270(01)02587-4> [PubMed](http://www.ncbi.nlm.nih.gov/entrez/query.fcgi?cmd=Retrieve&db=PubMed&list_uids=11510775&dopt=Abstract)

29. Crow WT, Willis DR. Estimating cost of care for patients with acute low back pain: a retrospective review of patient records. J Am Osteopath Assoc 2009;109(4):229–33. [PubMed](http://www.ncbi.nlm.nih.gov/entrez/query.fcgi?cmd=Retrieve&db=PubMed&list_uids=19369510&dopt=Abstract)

30. Younossi ZM, Zheng L, Stepanova M, Henry L, Venkatesan C, Mishra A. Trends in outpatient resource utilizations and outcomes for Medicare beneficiaries with nonalcoholic fatty liver disease. J Clin Gastroenterol 2015;49(3):222–7. <http://dx.doi.org/10.1097/MCG.0000000000000071> [PubMed](http://www.ncbi.nlm.nih.gov/entrez/query.fcgi?cmd=Retrieve&db=PubMed&list_uids=24637730&dopt=Abstract)

31. Riley GF, Lubitz JD. Long-term trends in Medicare payments in the last year of life. Health Serv Res 2010;45(2):565–76. <http://dx.doi.org/10.1111/j.1475-6773.2010.01082.x> [PubMed](http://www.ncbi.nlm.nih.gov/entrez/query.fcgi?cmd=Retrieve&db=PubMed&list_uids=20148984&dopt=Abstract)

# Appendix 2. Detailed Model Outcomes

## Table 1. Simulated Outcomes – Prediabetes Population Total

| Outcomes | Without Intervention | | | With Intervention | | | Intervention Impact | | |
| --- | --- | --- | --- | --- | --- | --- | --- | --- | --- |
|  | 3-year | 5-year | 10-year | 3-year | 5-year | 10-year | 3-year | 5-year | 10-year |
| **Disease onset** **(%)** | | | | | | | | | |
| Diabetes | 24.4 | 38.1 | 62.7 | 17.6 | 26.8 | 46.4 | −6.8 | −11.2 | −16.3 |
| History of ischemic heart disease | 2.0 | 3.9 | 10.8 | 1.8 | 3.4 | 9.2 | −0.2 | −0.5 | −1.5 |
| History of myocardial infarction | 1.2 | 2.3 | 6.8 | 1.0 | 2.1 | 5.7 | −0.1 | −0.3 | −1.1 |
| History of congestive heart failure | 3.4 | 6.4 | 16.5 | 2.9 | 5.4 | 14.0 | −0.5 | −1.0 | −2.5 |
| History of stroke | 2.4 | 4.8 | 12.7 | 2.1 | 4.0 | 10.7 | −0.4 | −0.8 | −2.0 |
| Obstructive sleep apnea | 16.4 | 28.1 | 59.1 | 14.9 | 25.3 | 53.4 | −1.6 | −2.8 | −5.8 |
| Major depression episodes | 23.5 | 40.1 | 84.0 | 21.4 | 36.1 | 74.9 | −2.1 | −4.0 | −9.2 |
| **Medical expenditures saving, $ per capita** | 22,855 | 41,738 | 102,004 | 21,545 | 38,873 | 92,787 | −1,310 | −2,865 | −9,217 |
| **QALYs increase per person** | 2.12 | 3.42 | 6.29 | 2.17 | 3.51 | 6.52 | 0.05 | 0.10 | 0.23 |
| **Sick days reduction per person** | 12.0 | 20.3 | 41.8 | 11.6 | 19.6 | 40.2 | −0.4 | −0.7 | −1.6 |
| **Average 3-year program costs, $** | 1,942 | 3,214 | 6,171 | 1,881 | 3,095 | 5,935 | −61 | −119 | −236 |
| **Average weight loss, %** | 0 | | | 1,300 | | | NA | | |
| **Sample size, n** | 0 | | | 5.1 | | |  |  |  |
| **Medical expenditures saving, $ per capita** | 1,663 | | | 1,663 | | |  |  |  |

Abbreviation: NA, not applicable; QALYs, quality-adjusted life years.

Note: Dollar estimates are 2014 US dollar values using 3% discount rate.

## Table 2. Simulated Outcomes – Prediabetes Population Starters

| Outcomes | Without Intervention | | | With Intervention | | | Intervention Impact | | |
| --- | --- | --- | --- | --- | --- | --- | --- | --- | --- |
|  | 3-year | 5-year | 10-year | 3-year | 5-year | 10-year | 3-year | 5-year | 10-year |
| **Disease onset** **(%)** | | | | | | | | | |
| Diabetes | 24.5 | 38.2 | 62.7 | 16.8 | 25.8 | 45.2 | −7.7 | −12.4 | −17.5 |
| History of ischemic heart disease | 2.0 | 3.9 | 10.9 | 1.8 | 3.5 | 9.6 | −0.2 | −0.5 | −1.3 |
| History of myocardial infarction | 1.2 | 2.4 | 7.0 | 1.1 | 2.1 | 5.9 | −0.1 | −0.3 | −1.1 |
| History of congestive heart failure | 3.5 | 6.6 | 16.9 | 3.0 | 5.6 | 14.4 | −0.5 | −1.0 | −2.5 |
| History of stroke | 2.5 | 4.8 | 13.0 | 2.1 | 4.1 | 11.0 | −0.4 | −0.8 | −2.0 |
| Obstructive sleep apnea | 16.6 | 28.4 | 60.0 | 14.7 | 25.4 | 53.8 | −1.9 | −3.1 | −6.2 |
| Major depression episodes | 23.5 | 40.1 | 84.0 | 20.9 | 35.5 | 73.6 | −2.6 | −4.6 | −10.4 |
| **Medical expenditures saving, $ per capita** | 23,329 | 42,597 | 104,488 | 21,796 | 39,280 | 94,444 | −1,533 | −3,317 | −10,043 |
| **QALYs increase per person** | 2.12 | 3.41 | 6.28 | 2.18 | 3.52 | 6.53 | 0.06 | 0.11 | 0.26 |
| **Sick days reduction per person** | 11.9 | 20.3 | 41.6 | 11.5 | 19.5 | 39.6 | −0.4 | −0.8 | −2.0 |
| **Average 3-year program costs, $** | 1,942 | 3,208 | 6,136 | 1,872 | 3,075 | 5,842 | −71 | −132 | −295 |
| **Average weight loss, %** | 0 | | | 1,300 | | | NA | | |
| **Sample size, n** | 0 | | | 5.3 | | |  |  |  |
| **Medical expenditures saving, $ per capita** | 1,462 | | | 1,462 | | |  |  |  |

Abbreviation: NA, not applicable; QALYs, quality-adjusted life years.

Note: Dollar estimates are 2014 US dollar values using 3% discount rate.

## Table 3. Simulated Outcomes – Prediabetes Population Completers

| Outcomes | Without Intervention | | | With Intervention | | | Intervention Impact | | |
| --- | --- | --- | --- | --- | --- | --- | --- | --- | --- |
|  | 3-year | 5-year | 10-year | 3-year | 5-year | 10-year | 3-year | 5-year | 10-year |
| **Disease onset** **(%)** | | | | | | | | | |
| Diabetes | 24.3 | 38.1 | 62.5 | 16.2 | 24.3 | 43.0 | −8.1 | −13.8 | −19.5 |
| History of ischemic heart disease | 2.3 | 4.3 | 11.9 | 1.9 | 3.7 | 10.2 | −0.4 | −0.7 | −1.7 |
| History of myocardial infarction | 1.4 | 2.6 | 7.6 | 1.1 | 2.2 | 6.1 | −0.2 | −0.5 | −1.5 |
| History of congestive heart failure | 3.7 | 6.9 | 17.9 | 3.1 | 5.7 | 14.8 | −0.6 | −1.1 | −3.2 |
| History of stroke | 2.7 | 5.2 | 13.7 | 2.1 | 4.1 | 11.2 | −0.6 | −1.1 | −2.6 |
| Obstructive sleep apnea | 17.0 | 29.1 | 61.2 | 15.0 | 25.6 | 54.3 | −2.0 | −3.5 | −7.0 |
| Major depression episodes | 22.7 | 38.8 | 81.5 | 20.0 | 33.5 | 69.8 | −2.7 | −5.3 | −11.8 |
| **Medical expenditures saving, $ per capita** | 23,829 | 43,643 | 107,879 | 22,039 | 39,749 | 95,853 | −1,790 | −3,893 | −12,026 |
| **QALYs increase per person** | 2.12 | 3.41 | 6.25 | 2.19 | 3.54 | 6.54 | 0.06 | 0.12 | 0.28 |
| **Sick days reduction per person** | 11.7 | 19.9 | 40.5 | 11.2 | 19.1 | 38.7 | −0.5 | −0.8 | −1.8 |
| **Average 3-year program costs, $** | 1,920 | 3,172 | 6,021 | 1,841 | 3,044 | 5,733 | −79 | −128 | −287 |
| **Average weight loss, %** | 0 | | | 1,300 | | | NA | | |
| **Sample size, n** | 0 | | | 6.0 | | |  |  |  |
| **Medical expenditures saving, $ per capita** | 1,232 | | | 1,232 | | |  |  |  |

Abbreviation: NA, not applicable; QALYs, quality-adjusted life years.

Note: Dollar estimates are 2014 US dollar values using 3% discount rate.

## Table 4. Simulated Outcomes – USPSTF Population Total

| Outcomes | Without Intervention | | | With Intervention | | | Intervention Impact | | |
| --- | --- | --- | --- | --- | --- | --- | --- | --- | --- |
|  | 3-year | 5-year | 10-year | 3-year | 5-year | 10-year | 3-year | 5-year | 10-year |
| **Disease onset** **(%)** | | | | | | | | | |
| Diabetes | 13.1 | 21.1 | 37.9 | 9.1 | 14.2 | 26.4 | −3.9 | −6.9 | −11.5 |
| History of ischemic heart disease | 2.2 | 4.1 | 11.2 | 1.9 | 3.7 | 10.2 | −0.2 | −0.4 | −1.0 |
| History of myocardial infarction | 1.3 | 2.3 | 6.5 | 1.1 | 2.1 | 5.6 | −0.2 | −0.2 | −0.9 |
| History of congestive heart failure | 3.6 | 6.6 | 16.5 | 3.3 | 6.0 | 14.8 | −0.3 | −0.6 | −1.7 |
| History of stroke | 2.5 | 4.8 | 12.8 | 2.2 | 4.3 | 11.2 | −0.3 | −0.5 | −1.7 |
| Obstructive sleep apnea | 15.7 | 26.8 | 57.0 | 13.8 | 23.7 | 50.8 | −1.9 | −3.1 | −6.2 |
| Major depression episodes | 29.7 | 50.0 | 103.7 | 26.8 | 45.4 | 92.9 | −2.9 | −4.6 | −10.9 |
| **Medical expenditures saving, $ per capita** | 24,773 | 44,505 | 105,906 | 23,377 | 41,693 | 97,954 | −1,396 | −2,812 | −7,951 |
| **QALYs increase per person** | 2.08 | 3.37 | 6.25 | 2.14 | 3.47 | 6.47 | 0.05 | 0.1 | 0.21 |
| **Sick days reduction per person** | 12.3 | 20.8 | 42.8 | 11.8 | 20.1 | 41.0 | −0.5 | −0.7 | −1.8 |
| **Average 3-year program costs, $** | 1,966 | 3,237 | 6,215 | 1,893 | 3,125 | 5,947 | −73 | −112 | −269 |
| **Average weight loss, %** | 0 | | | 1,300 | | | NA | | |
| **Sample size, n** | 0 | | | 5.0 | | |  |  |  |
| **Medical expenditures saving, $ per capita** | 2,152 | | | 2,152 | | |  |  |  |

Abbreviation: NA, not applicable; QALYs, quality-adjusted life years.

Note: Dollar estimates are 2014 US dollar values using 3% discount rate.

## Table 5. Simulated Outcomes – USPSTF Population Starters

| Outcomes | Without Intervention | | | With Intervention | | | Intervention Impact | | |
| --- | --- | --- | --- | --- | --- | --- | --- | --- | --- |
|  | 3-year | 5-year | 10-year | 3-year | 5-year | 10-year | 3-year | 5-year | 10-year |
| **Disease onset** **(%)** | | | | | | | | | |
| Diabetes | 13.0 | 20.9 | 37.7 | 8.7 | 13.5 | 25.5 | −4.3 | −7.3 | −12.2 |
| History of ischemic heart disease | 2.2 | 4.2 | 11.7 | 2.0 | 3.8 | 10.2 | −0.2 | −0.5 | −1.5 |
| History of myocardial infarction | 1.2 | 2.4 | 6.7 | 1.1 | 2.1 | 5.7 | −0.1 | −0.3 | −0.9 |
| History of congestive heart failure | 3.5 | 6.6 | 16.8 | 3.3 | 5.9 | 14.7 | −0.2 | −0.7 | −2.1 |
| History of stroke | 2.6 | 5.0 | 13.1 | 2.3 | 4.3 | 11.5 | −0.3 | −0.6 | −1.6 |
| Obstructive sleep apnea | 15.9 | 27.1 | 57.5 | 14.1 | 24.1 | 51.2 | −1.8 | −3.0 | −6.3 |
| Major depression episodes | 29.1 | 49.2 | 102.2 | 26.1 | 43.9 | 90.0 | −3.1 | −5.3 | −12.1 |
| **Medical expenditures saving, $ per capita** | 25,077 | 45,092 | 107,669 | 23,508 | 42,045 | 99,059 | −1,569 | −3,047 | −8,610 |
| **QALYs increase per person** | 2.09 | 3.38 | 6.25 | 2.15 | 3.48 | 6.48 | 0.06 | 0.11 | 0.23 |
| **Sick days reduction per person** | 12.2 | 20.7 | 42.2 | 11.7 | 19.9 | 40.5 | −0.5 | −0.8 | −1.7 |
| **Average 3-year program costs, $** | 1,961 | 3,222 | 6,134 | 1,881 | 3,096 | 5,888 | −80 | −126 | −246 |
| **Average weight loss, %** | 0 | | | 1,300 | | | NA | | |
| **Sample size, n** | 0 | | | 5.3 | | |  |  |  |
| **Medical expenditures saving, $ per capita** | 1,892 | | | 1,892 | | |  |  |  |

Abbreviation: NA, not applicable; QALYs, quality-adjusted life years.

Note: Dollar estimates are 2014 US dollar values using 3% discount rate.

## Table 6. Simulated Outcomes – USPSTF Population Completers

| Outcomes | Without Intervention | | | With Intervention | | | Intervention Impact | | |
| --- | --- | --- | --- | --- | --- | --- | --- | --- | --- |
|  | 3-year | 5-year | 10-year | 3-year | 5-year | 10-year | 3-year | 5-year | 10-year |
| **Disease onset** **(%)** | | | | | | | | | |
| Diabetes | 11.1 | 17.8 | 31.6 | 7.2 | 11.0 | 20.3 | −3.9 | −6.7 | −11.3 |
| History of ischemic heart disease | 2.4 | 4.6 | 12.3 | 2.1 | 4.0 | 10.7 | −0.3 | −0.6 | −1.6 |
| History of myocardial infarction | 1.3 | 2.5 | 7.1 | 1.2 | 2.2 | 5.9 | −0.2 | −0.3 | −1.2 |
| History of congestive heart failure | 3.9 | 7.0 | 17.7 | 3.4 | 6.3 | 15.5 | −0.5 | −0.8 | −2.2 |
| History of stroke | 2.7 | 5.1 | 13.7 | 2.2 | 4.3 | 11.6 | −0.5 | −0.8 | −2.1 |
| Obstructive sleep apnea | 16.1 | 27.6 | 58.6 | 14.4 | 24.7 | 52.5 | −1.8 | −2.8 | −6.0 |
| Major depression episodes | 27.7 | 46.6 | 97.1 | 24.3 | 41.1 | 84.3 | −3.5 | −5.5 | −12.8 |
| **Medical expenditures saving, $ per capita** | 25,628 | 46,221 | 111,294 | 23,863 | 42,785 | 101,328 | −1,765 | −3,436 | −9,966 |
| **QALYs increase per person** | 2.09 | 3.38 | 6.23 | 2.15 | 3.49 | 6.49 | 0.07 | 0.12 | 0.26 |
| **Sick days reduction per person** | 12.0 | 20.3 | 41.5 | 11.5 | 19.4 | 39.4 | −0.5 | −0.9 | −2.1 |
| **Average 3-year program costs, $** | 1,931 | 3,182 | 6,061 | 1,856 | 3,042 | 5,752 | −75 | −140 | −309 |
| **Average weight loss, %** | 0 | | | 1,300 | | | NA | | |
| **Sample size, n** | 0 | | | 6.0 | | |  |  |  |
| **Medical expenditures saving, $ per capita** | 1,588 | | | 1,588 | | |  |  |  |

Abbreviation: NA, not applicable; QALYs, quality-adjusted life years.

Note: Dollar estimates are 2014 US dollar values using 3% discount rate.

# Appendix 3. Sensitivity Analysis, USPSTF Population

## Appendix Figure 2. Sensitivity analysis on weight loss over 10 years, USPSTF population.


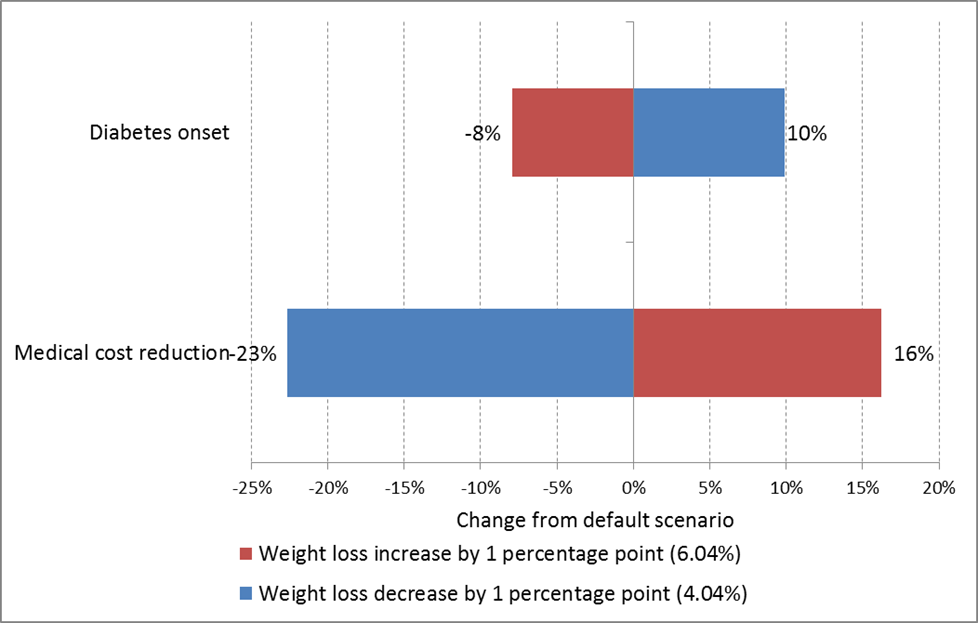


Note: default weight loss for USPSTF population is 5.04%; diabetes onset is based on the absolute number of new diabetes cases in the time period; a tornado diagram shows how key outcomes change relative to default scenario. The red or blue bars above represent the change in model outcome when weight loss increased or decreased by 1 percentage point, respectively.
